# Comparison Between Ultrasonographic and Standing Magnetic Resonance Imaging Findings in the Podotrochlear Apparatus of Horses With Foot Pain

**DOI:** 10.3389/fvets.2021.675180

**Published:** 2021-07-05

**Authors:** Laurence Evrard, Zoë Joostens, Maxime Vandersmissen, Fabrice Audigié, Valeria Busoni

**Affiliations:** ^1^Department of Clinical Sciences of Companion Animals and Equids, Equine Division, Diagnostic Imaging Section, University of Liège, Liège, Belgium; ^2^Medical Imaging Department, Equitom Equine Clinic, Lummen, Belgium; ^3^Centre d'Imagerie et de Recherche sur les Affections Locomotrices Equines, Ecole Nationale Vétérinaire d'Alfort, Goustranville, France

**Keywords:** equine, MRI, ultrasound, foot pain, navicular syndrome

## Abstract

This prospective study aimed to blindly compare the ultrasonographic and standing magnetic resonance imaging (sMRI) findings in deep digital flexor tendon (DDFT), navicular bone, and navicular bursa in horses with foot pain, positive digital analgesia, and without definitive radiographic diagnosis. Ultrasonography detected more DDFT abnormalities (32/34 feet vs. 27/34 with sMRI) but identified less palmar navicular abnormalities (23/34 feet vs. 30/34 with sMRI). In suprasesamoidean DDFT lesions, which were mainly dorsally located, changes in echogenicity did not correspond to a particular pattern of sMRI signal change. Transcuneal ultrasonography did not allow assessment of morphology and extent of distal DDFT lesions, and sporadically discriminated the affected lobe compared to sMRI. Defects of the palmar compact bone were identified with both modalities except a parasagittal defect, which was only seen at sMRI.

## Introduction

Foot pain is a common cause of lameness in horses, and imaging is necessary to reach a definitive and detailed diagnosis (1).

Ultrasonography is an easily available and cost-effective diagnostic technique allowing to assess the podotrochlear apparatus in the distal pastern, through the bulbs of the heels (2–4) and by transcuneal approach (5–11). The pastern approach allows detection of lesions of the deep digital flexor tendon (DDFT), navicular bursa, collateral sesamoidean ligament (CSL), and distal digital annular ligament (DDAL) (2–4, 12). The transcuneal approach allows detection of irregularities of the flexor surface of the navicular bone (6–8, 12) and abnormalities of the distal aspect of the DDFT, as well as changes in the distal sesamoidean impar ligament (DSIL) and their enthesis (3, 6, 9, 12). Transcuneal ultrasonography has been compared to post-mortem for lesion detection (7) and demonstrated a good sensitivity for detection of irregularities of the flexor surface. However, it is limited by the frog conformation and hydration, and does not allow evaluation of the abaxial portions of the tendon because of the sagittal acoustic window given by the frog (5). Ultrasonographic abnormalities have been reported in horses without significant radiographic changes at the radiographic examination using both distal palmar pastern and transcuneal approaches, and ultrasonography has been suggested as a complementary modality for soft tissue assessment in the foot in cases where financial or geographical reasons impair the use of standing magnetic resonance imaging (sMRI) (3). Ultrasonography is also routinely used to guide injections in the foot area, and the use of both approaches has been described (13–15).

Magnetic resonance imaging (MRI) of the equine foot has largely been addressed in literature as the most comprehensive technique to explore the causes of foot pain, involving various deep structures within the foot (16–23). Magnetic resonance imaging has been helpful to classify DDFT lesions in core, dorsal lesions, and tendon splits (16, 18, 24–26) and to accurately document that DDFT lesions may occur alone or in combination with other soft tissue or bone abnormalities in the foot (18, 20, 25). Previous reports have highlighted the importance of MRI to identify edema-like lesions in the navicular bone in horses without radiographic changes (21) and its ability to well-describe erosive lesions of the palmar compact bone (27). Both high-field and low-field MRI are available for diagnostic procedures in equine patients (28, 29), although the use of low-field MRI has increased following the emergence of a specific system allowing to examine standing patients under simple sedation (sMRI) (22). Publications have compared image quality (28) and accuracy of lesion detection (29) between both systems, and agreement with histopathology is considered acceptable for diagnostic purposes for both (30, 31). Standing magnetic resonance imaging has lower spatial resolution compared with low or high-field systems used under general anesthesia, due to motion artifact, although this would more severely impact acquisitions of the proximal limb (32).

Whereas, ultrasonography and MRI findings in equine feet have been largely independently reported, direct comparison between the appearance of lesions with both modalities has only sporadically been done (12). This study aimed to compare sMRI and ultrasonographic findings in the podotrochlear apparatus in horses with foot pain without relevant radiological findings. We hypothesized that:

- suprasesamoidean DDFT lesions and abnormalities of the flexor surface of the navicular bone would be visible both at sMRI and ultrasonography using a palmar approach through the bulbs of the heels;
- sesamoidean DDFT lesions, parasagittal DDFT splits (whatever the location), and lesions of the navicular spongiosa visible at sMRI would not be identified at ultrasonography;
- infrasesamoidean DDFT lesions visible at sMRI would be seen at transcuneal ultrasonography only in case of tendon thickening causing convex deformity of the palmar tendon profile.

## Materials and Methods

### Study Design and Horse Selection

This original research investigation is a prospective descriptive study. Horses referred for forelimb lameness between 2016 and 2019, responding to distal digital or sesamoidean abaxial analgesia, were prospectively included following owner consent, if radiographs were considered inconclusive and if blind examination by the two principal investigators was possible in the clinical settings. Horses with radiographic abnormalities of the podotrochlear apparatus with potential clinical significance and in particular palmar compact erosions, osseous cyst-like lesions, or fracture of the navicular bone visible on radiographs were excluded. Horses referred for sMRI and with radiographic abnormalities in the foot other than in the podotrochlear apparatus and considered clinically significant and horses with history of trauma or sepsis were also not included.

### Standing MRI Material and Protocol

Standing magnetic resonance imaging examination of the foot was performed using a standing low-field system (Hallmarq, Guildford, UK) with a hoof dedicated coil. Horses were sedated with intravenous acepromazine premedication (0.05 mg/kg), followed by a bolus of detomidine (0.01 mg/kg) and morphine (0.1 mg/kg). Horses were maintained under sedation during the MRI examination using a continuous drip of morphine (0.05 mg/kg) and detomidine (0.022 mg/kg) in NaCl (0.9%). Standing magnetic resonance imaging examinations were performed using a two-step protocol: a basic standard protocol was acquired in all horses including tridimensional gradient echo T1-weighted (T1W 3D) images in the three planes (sagittal, dorsal, and transverse), short tau inversion recovery fast spin echo weighted (STIR FSE) images in the sagittal plane, and T2-weighted fast spin echo (T2W FSE) images in the transverse plane. The transverse and dorsal T1W 3D sequences were high-resolution sequences except when prevented by motion. Transverse images were angled perpendicular to the suprasesamoidean portion of the DDFT or to the navicular bone flexor surface, or both planes were acquired depending on the visualized lesion(s). The T2W FSE transverse sequence was either a standard either a high resolution sequence, depending on the patient compliance, with the transverse plane oriented perpendicular to the suprasesamoidean DDFT. In addition to the standard basic protocol, an additional transverse T2W FSE sequence (either perpendicular to the infrasesamoidean DDFT or parallel to the sole), a transverse tridimensional out of phase T2^*^ weighted (T2^*^oW 3D), and an additional transverse and/or dorsal STIR sequences were acquired depending on the findings observed at the time of the acquisition by the radiologist performing the examination. The pastern region was not examined at sMRI. Standing magnetic resonance imaging parameters are summarized in Table 1.

**Table 1:** Standing magnetic resonance acquisition parameters.

**Sequence**	**Slice thickness (mm)**	**Field of view (mm)**	**Matrix**	**TE (s)**	**TR (s)**	**Gap**	**Scan time**
T1 3D	2.97	180	256*256	7	24	0	2 min 25 s
T2*oW 3D	2.97	180	256*256	13	34	0	2 min3 4 s
T2 FSE	5	180	256*256	81	1,848	1	3 min 29 s
STIR FSE	5	180	256*256	22	2,576	1	5 min 18 s
T1 3D HR	1.48	180	512*512	8	24	0	3 min 57 s
T2 FSE HR	5	180	512*512	87	1,815	1	5 min 42 s
STIR FSE HR	5	180	512*512	29	2,316	1	5 min 20 s

*3D, tridimensional gradient echo; oW, out of phase weighted; STIR, short tau inversion recovery, FSE, fast spin echo; HR, high resolution; TE, time of echo; TR, time of repetition*.

### Ultrasonographic Material and Protocol

**Figure 1:**
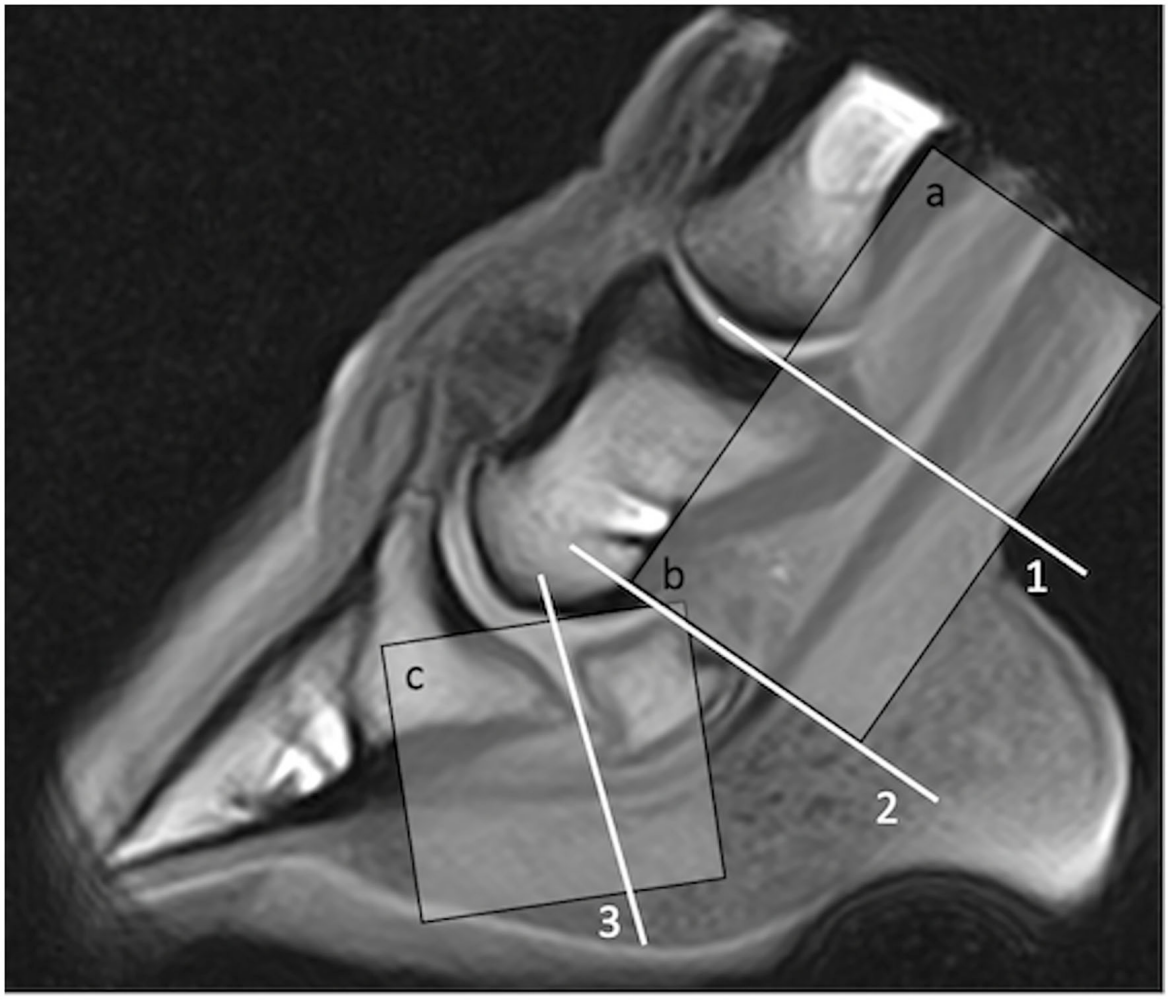
Proximo-distal levels used to describe deep digital flexor tendon lesion location at magnetic resonance (MR) imaging (1, suprasesamoidean; 2, sesamoidean; 3, infrasesamoidean) and at ultrasonography (a, pastern; b, suprasesamoidean; c; distal sesamoidean and infrasesamoidean explored together with transcuneal approach). Region a (pastern) was not imaged using MR in this study.

After sMRI examination, the pastern was clipped and the skin was prepared for ultrasonographic examination. The frog was trimmed to obtain a flat surface and to increase the available acoustic window between the apex of the frog and the central sulcus. A sponge wet with warm water was placed under the frog to soften the horn. Ultrasonographic examinations were performed using a high-resolution linear and curvilinear (microconvex) transducer (1–15 MHz; Hitachi Aloka F37, Steinhausen, Germany), using palmar and transcuneal approaches (2, 7). Images of the suprasesamoidean portion of the DDFT were obtained *via* the palmar approach of the distal pastern through the bulbs of the heels on the weight-bearing limb with the curvilinear probe, whereas images of the distal aspect of the navicular bone flexor surface, distal sesamoidean, and infrasesamoidean portions of the DDFT were obtained *via* the transcuneal approach through the frog on the non-weight-bearing limb using the linear transducer (Figure 1). Frequency was lowered to better penetrate the horn through the frog, and focus was placed at the level of the navicular bone surface to optimize lateral resolution. Mid and proximal pastern examination using the linear transducer was done to explore the proximal extent of suprasesamoidean DDFT lesions. Power Doppler was used on transverse images to assess DDFT lesion vascularisation in the suprasesamoidean area using the same transducer as for B-mode examination, with the non-weight-bearing limb in a flexed position. Sensitivity was optimized for low flow, the lowest possible pulse repetition frequency, the lowest possible wall filter, and color gain set just below the noise level.

### Image Acquisition and Analysis

#### Image Acquisition and Recording of Abnormalities

Standing magnetic resonance imaging examinations and ultrasonographic examinations were performed independently by two board-certified radiologists (L.E. and V.B., respectively), each blinded to the results of the other imaging examination. Therefore, all horses responding to inclusion criteria underwent a complete examination in both modalities. Ultrasonographic and sMRI findings were subsequently blindly recorded and independently analyzed by the board-certified radiologists (V.B. and L.E.). Only images of the podotrochlear apparatus were used and navicular bone, DDFT, and navicular bursa were assessed for ultrasonographic and sMRI abnormalities, according to previously described criteria (3, 4, 17, 21, 24, 25, 33).

#### Lesion Classification Criteria

Lesions of the DDFT were classified following their location at ultrasonography and sMRI (Figure 1). At ultrasonography, they were characterized as tendon lobe thickening or deformity (dorsal vs. palmar), with or without change in echogenicity (hypo-, iso-, or hyperechoic to the remainder of the tendon lobe). Lesions were reported as present or absent, without grading severity. Doubtful lesions were reported as “equivocal.” At sMRI, DDFT lesions were characterized following their morphology, defining lesions as dorsal, core lesions, or parasagittal splits if they were, respectively, affecting the dorsal border of the lobe, the center of the lobe, or if they were crossing through the lobe with parasagittal location (17, 21, 24, 33). Standing magnetic resonance imaging lesions were classified as mild in case of dorsal irregularity/deformity without change in signal in the adjacent deeper part or in case of slight and focal GRE hyperintensity, with or without corresponding T2 and STIR FSE hyperintensity. They were considered moderate if a hyperintense signal was clearly visible in GRE without exceeding half of the lobe thickness in cross-section neither exceeding 30 mm in proximo-distal length, with or without corresponding T2 and STIR FSE hyperintensity. Based on previous results about DDFT lesions prognosis, lesions were considered severe if they exceeded 30 mm in proximo-distal length (34) and/or exceeded half of the lobe thickness in cross-section in GRE images, and if they were visible both in GRE and T2 and STIR FSE sequences.

Lesions of the flexor surface of the navicular bone were characterized as erosive when a palmar compact defect was identified with both modalities (7, 27). Irregularity of the flexor surface without compact defect was also reported on both modalities, as well as subjective thinning and change in signal of the palmar fibrocartilage in sMRI and change in signal of the palmar spongiosa.

Bursitis was reported at ultrasonography and sMRI when the navicular bursa was effused, resulting in an increased size of the proximal recess in comparison to reference images, with or without soft tissue material and/or mesotendon thickening (3, 25).

#### Image Comparative Analysis

Ultrasonographic and sMRI lesions recorded by each operator independently at the time of image acquisition were subsequently compared in consensus between the first and last authors (L.E. and V.B.). DDFT lesions were compared for location, shape, and signal intensity at sMRI vs. echogenicity at ultrasonography. Lesion size was not compared since ultrasonography was not considered reliable in measuring the proximodistal extent of a lesion. Navicular bone flexor surface lesions were compared for the presence of an erosive lesion or irregularity of the flexor surface with or without changes in fibrocartilage at sMRI. Navicular bursa abnormalities were compared for the presence of effusion with or without increased soft tissue material in the proximal recess.

### Statistics

Percentage of agreement and Cohen's kappa (k) were calculated by the last author (V.B.) using an on-line calculator (http://vassarstats.net/kappa.html) to evaluate agreement between ultrasonography and sMRI for the suprasesamoidean and distal (sesamoidean and infrasesamoidean) DDFT lesions, lesions of the palmar compact of the navicular bone, and navicular bursitis. Because the kappa coefficient is influenced by the prevalence of each abnormality and the extent to which the raters disagree on the proportion of positive (or negative) cases, prevalence-adjusted and bias-adjusted kappa (PABAk) were also manually calculated based on the formulae reported in the literature (35, 36) to evaluate the effect of prevalence and bias index on k.

## Results

### Horses, Feet, and Imaging Examination

Thirty-seven feet from 31 horses fulfilled the selection criteria. Three feet were excluded because the sMRI examination was considered insufficient (excessive motion or shortened protocol due to lack of patient compliance). Thirty-four feet from 30 horses were then used (17 left front feet, 17 right front feet).

Horses included were 14 mares, 13 geldings, and 3 stallions, aging from 5 to 16 years old (mean 11 years old, median 11 years old). Twenty-seven horses were Warmblood horses, two were Arabian, and one was a Standardbred. Horses were mainly pleasure horses or low-level show-jumping or dressage horses.

In three feet, the transverse T1W 3D sequence had to be acquired as a standard sequence (matrix 256 × 256) instead of a high-resolution sequence (matrix 512 × 512), because of motion. A transverse STIR FSE sequence was acquired in 29 feet, while a dorsal STIR sequence was acquired in 4.

Transcuneal ultrasonographic images were not acquired in one foot, due to the inability to penetrate the frog with the ultrasonographic beam, but the foot was included for comparison of abnormalities recorded using the palmar approach through the bulbs of the heels.

### Imaging Abnormalities

#### DDFT

Abnormalities of the DDFT within the foot were observed in 30/34 feet at ultrasonography and in 27/34 feet at sMRI (Supplementary Table 1). Most DDFT abnormalities affected different levels (in 20 feet at sMRI and in 23 feet at ultrasonography), from the pastern (ultrasonography) or suprasesamoidean (sMRI) area to its distal enthesis. Both lobes of the DDFT were involved in 18 feet at sMRI and in 20 feet at ultrasonography.

##### Pastern

Twelve DDFT lesions were ultrasonographically detected in the proximal–mid pastern area, which was not assessed with sMRI. These were dorsal (6/12), core lesions (3/12), palmar (2/12), or axial (1/12 feet). These lesions were subsequently not compared.

##### Suprasesamoidean Level

**Figure 2:**
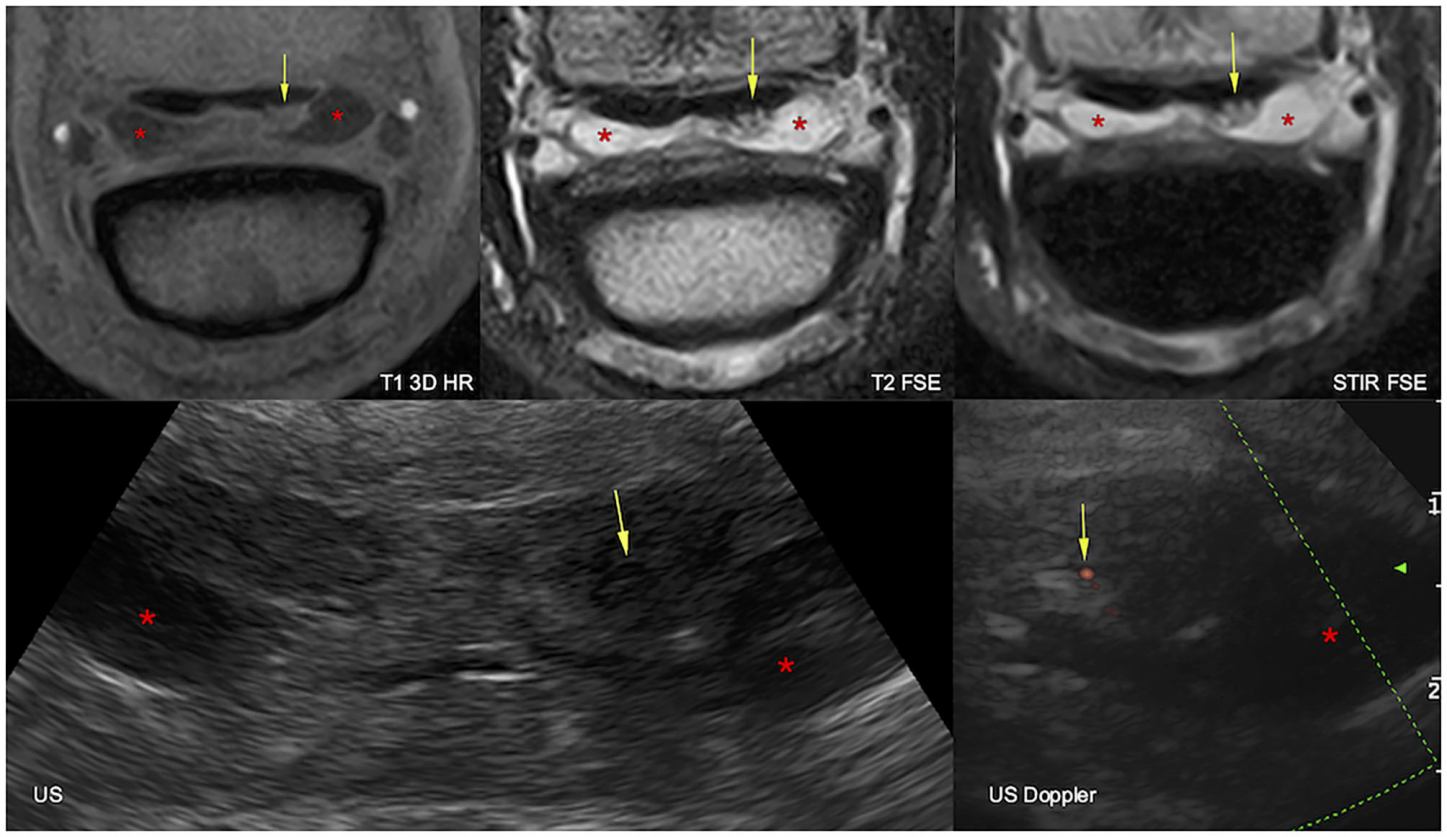
Transverse magnetic resonance imaging (MRI, top) and ultrasonographic (bottom) images of the right front foot at the suprasesamoidean level: lateral is to the right and dorsal to the bottom. The lateral lobe of the deep digital flexor tendon is increased in size and deformed by a dorsal lesion, hyperintense in all MRI sequences, hypoechoic, and vascularized at ultrasound (arrows). The navicular bursa is moderately effused (asterisks). T1 3D HR, high resolution tridimensional gradient echo T1 weighted sequence; T2 FSE, fast spin echo T2 weighted sequence; STIR FSE, fast spin echo short tau inversion recovery weighted sequence; US, ultrasonography; US Doppler, Doppler ultrasonography.

In the suprasesamoidean area, DDFT abnormalities were identified both at ultrasonography and sMRI in 22 feet (Figure 2), although in four biaxial lesions, there was discrepancy about the most affected lobe. In eight feet, suprasesamoidean abnormalities detected at ultrasonography were not visible at sMRI. Conversely, sMRI detected a dorsal lesion in one or both lobes of two feet, which was not visible at ultrasonography (Table 2). Doppler signal was visible at ultrasonography in three suprasesamoidean lesions and intratendinous mineralization in three, not visible at sMRI. At ultrasonography, lesions caused dorsal deformity of the tendon lobe in 27/30 feet and were hypoechoic (13 feet) or isoechoic (14 feet) to the remainder of the tendon. Overall lobe thickening was observed in two feet, and a full-thickness hyperechoic line was visible in the last foot. At sMRI, dorsal suprasesamoidean lesions were hyperintense in gradient echo images in 20/24 feet, and hyperintense in FSE-weighted sequences in 12 of those (all hyperintense in T2 FSE, and 10 hyperintense in STIR). In six feet, dorsal lesions were associated with central hyperintensity (core lesions) and/or with marked disruption of the dorsal tendinous border at sMRI. The four remaining suprasesamoidean lesions were characterized at sMRI by an irregular or deformed dorsal contour without major signal change. Changes in echogenicity did not correspond to a particular pattern of sMRI signal change. Percentage of agreement for detection of suprasesamoidean lesions was 70.6%. Cohen's kappa indicated only slight agreement (k = 0.14), but agreement was moderate when k was adjusted for prevalence and bias (PABAk = 0.41). Contingency tables and kappa values are presented in Supplementary File 2.

**Table 2:** Summary of suprasesamoidean lesions detected with one modality only.

**Suprasesamoidean DDFT lesions**	**Lesion morphology**	**Feet**
US lesions undetected with sMRI	Dorsal deformity	5
	*Isoechoic*	*4*
	*Hypoechoic*	*1*
	Thickening	2
	Full-thickness hyperechoic line	1
	**Total**	**8**
sMRI lesions undetected with US	Dorsal lesion	1
	Dorsal irregularity	1
	**Total**	**2**

*DDFT, deep digital flexor tendon; US, Ultrasonography; sMRI, standing magnetic resonance imaging*.

##### Sesamoidean/Infrasesamoidean Level

**Figure 3:**
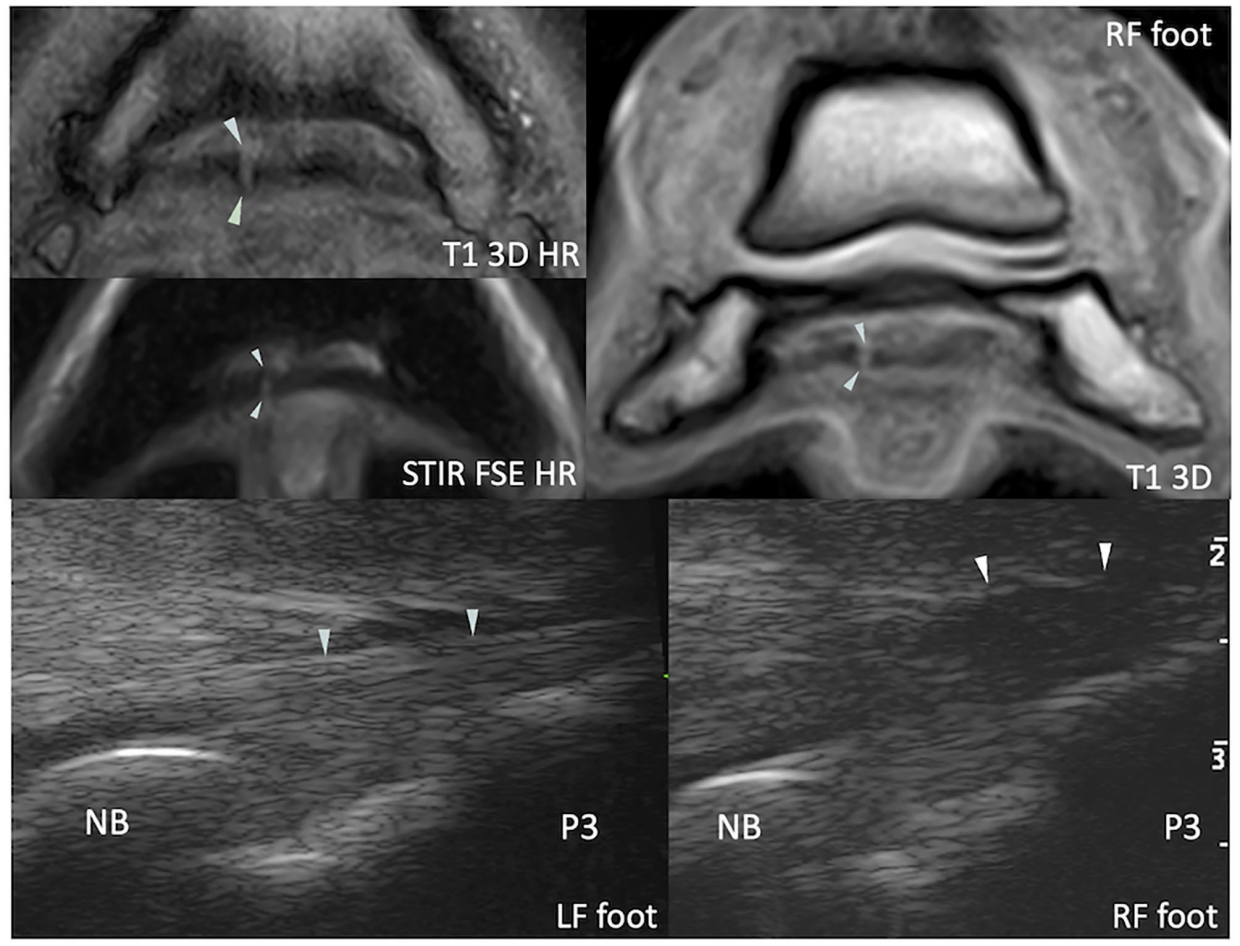
Transverse magnetic resonance (MR) images of the right front foot (top) and comparative transcuneal longitudinal ultrasonographic images of the left and right front feet (bottom) at the infrasesamoidean level. A hyperintense parasagittal tendon split is visible in the medial lobe of the deep digital flexor tendon on MR images (arrowheads). At ultrasound, the abnormal tendon (right front foot) appears thickened in its most distal portion and has a slightly convex palmar border (arrowheads). The tendon also appears hypoechoic in comparison to the contralateral foot, although this may be due to anisotropy and induced by a non-parallel fiber alignment to the ultrasound probe. T1 3D (HR), (high resolution) tridimensional gradient echo T1 weighted image; STIR FSE HR, high resolution fast spin echo short tau inversion recovery weighted sequence; NB, navicular bone; P3, distal phalanx.

Both ultrasonography and sMRI detected distal (sesamoidean/infrasesamoidean) DDFT abnormalities in 15 feet (Figure 3). Standing magnetic resonance imaging identified lesions in seven other feet where the tendon appeared normal at ultrasonography, while ultrasonography suspected tendon thickening in five feet, where sMRI did not identify any abnormality. At ultrasonography, the distal DDFT lesions (20 feet) were characterized by overall tendon thickening and/or palmar convexity without major change in echogenicity in all but one foot, where lateral hypoechogenicity was identified at the infrasesamoidean level. Sesamoidean lesions (17 feet) were seen at sMRI as an irregularly marginated dorsal border of one or both lobes (11 feet), tendon splits (5 feet), or core lesions (9 feet). Different types of lesions were found concurrently. Lesions were hyperintense in gradient echo images only in 11 feet, and in gradient echo and FSE-weighted sequences in six feet. Infrasesamoidean lesions detected at sMRI (twelve feet) were tendon splits (five feet), core lesions (five feet), or dorsal lesions (two feet). These lesions were hyperintense in gradient echo only in six feet, and in both gradient echo and FSE-weighted sequences in six feet. All but one infrasesamoidean lesions extended until the insertion on the distal phalanx at sMRI. Transcuneal ultrasonography did not allow assessment of morphology and extent of the distal tendon lesions, nor to discriminate the affected lobe (medial vs. lateral), except in three feet where lateral tendon thickening (*n* = 2) and hypoechogenicity (*n* = 1) corresponded to a lateral lobe lesion at sMRI. Percentage of agreement for detection of distal DDFT lesions was 63.6%. Cohen's kappa and PABAk indicated fair agreement (k = 0.22 and 0.27, respectively).

##### Lesion-Severity

At sMRI, lesions were considered mild in 5 feet, moderate in 12 feet, and severe in 10 feet. Ultrasonography detected 4/5 mild lesions, although there was discrepancy between both modalities about the affected lobe in two cases. Moderate sMRI lesions were detected with ultrasonography in 10/12 feet, including one vascularized suprasesamoidean lesion. There was discrepancy between both modalities about the most affected lobe in two feet with biaxial suprasesamoidean lesions. All 10 severe sMRI lesions were detected with ultrasonography, both at the suprasesamoidean and sesamoidean/infrasesamoidean levels. They included three lateralized infrasesamoidean lesions, two suprasesamoidean vascularized lesions, and two mineralized lesions (one suprasesamoidean and one infrasesamoidean) observed at ultrasonography (Table 3).

**Table 3:** Summary of deep digital flexor tendon lesion severity at standing magnetic resonance imaging and comparison with ultrasonographic findings.

**Lesion severity sMRI**	**Affected feet with sMRI**	**Affected feet with US**	**Lesion mineralization at US**	**Lesion vascularization at US**
Mild	5	4	0	0
Moderate	12	10	0	1
Severe	10	10	2	2

*sMRI, standing magnetic resonance imaging; US, ultrasonography*.

#### Navicular Bone

**Figure 4:**
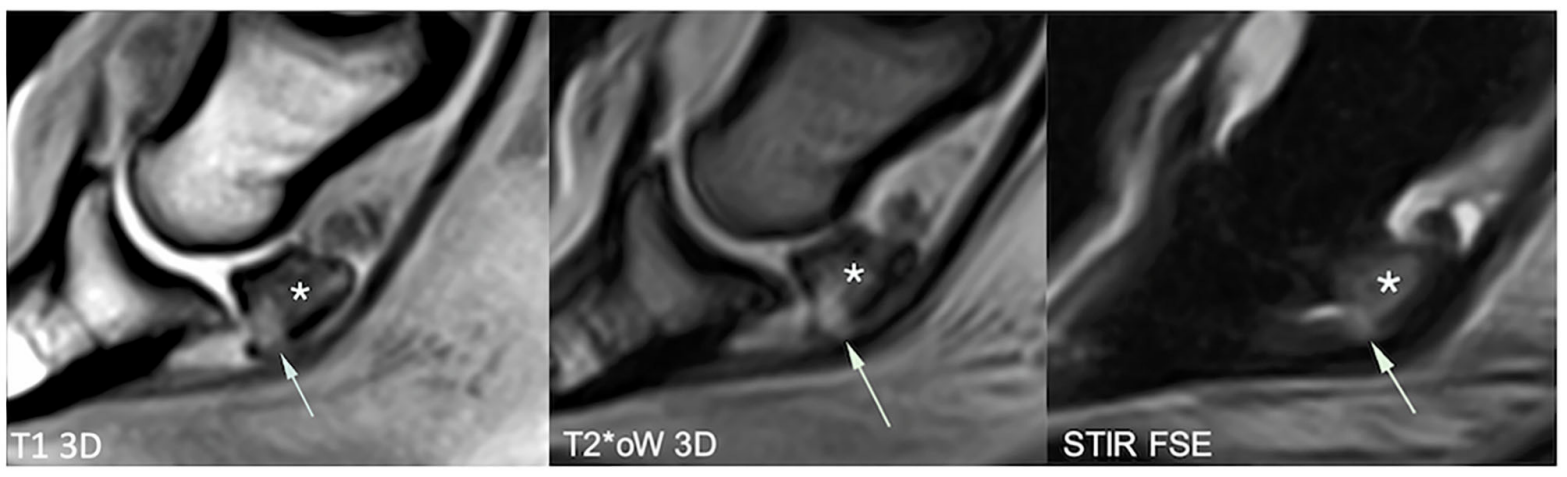
Sagittal magnetic resonance (MR) images focused on the navicular bone. There is a distal palmar compact bone erosion, characterized by disruption of the palmar compact and hyperintense signal intensity in all MR sequences, penetrating the adjacent palmar spongiosa (arrows). The navicular bone also shows extensive T1 hypointensity, STIR hyperintensity, and mixed T2*oW signal in the distal two-thirds of the palmar spongiosa, consistent with bone edema-like signal (asterisk). T1 3D, tridimensional gradient echo T1 weighted sequence; T2*oW 3D, tridimensional out of phase gradient echo T2* weighted sequence; STIR FSE, fast spin echo short tau inversion recovery weighted sequence; US, Ultrasonography.

**Figure 5:**
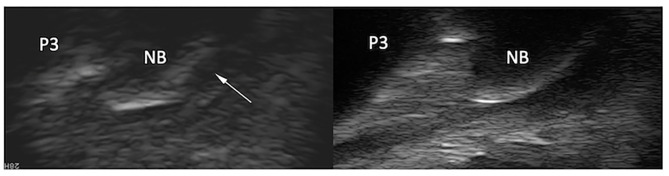
Longitudinal transcuneal ultrasonographic image of the same front foot than Figure 4 (left) and comparative image of a normal navicular bone (right). Only the distal aspect of the navicular bones is visible on the ultrasonographic images. The images have been flipped vertically to obtain the same orientation than the MR images. The erosive defect is characterized by disruption of the normal hyperechoic bone profile and deep penetrating echoes (arrow).

**Figure 6:**
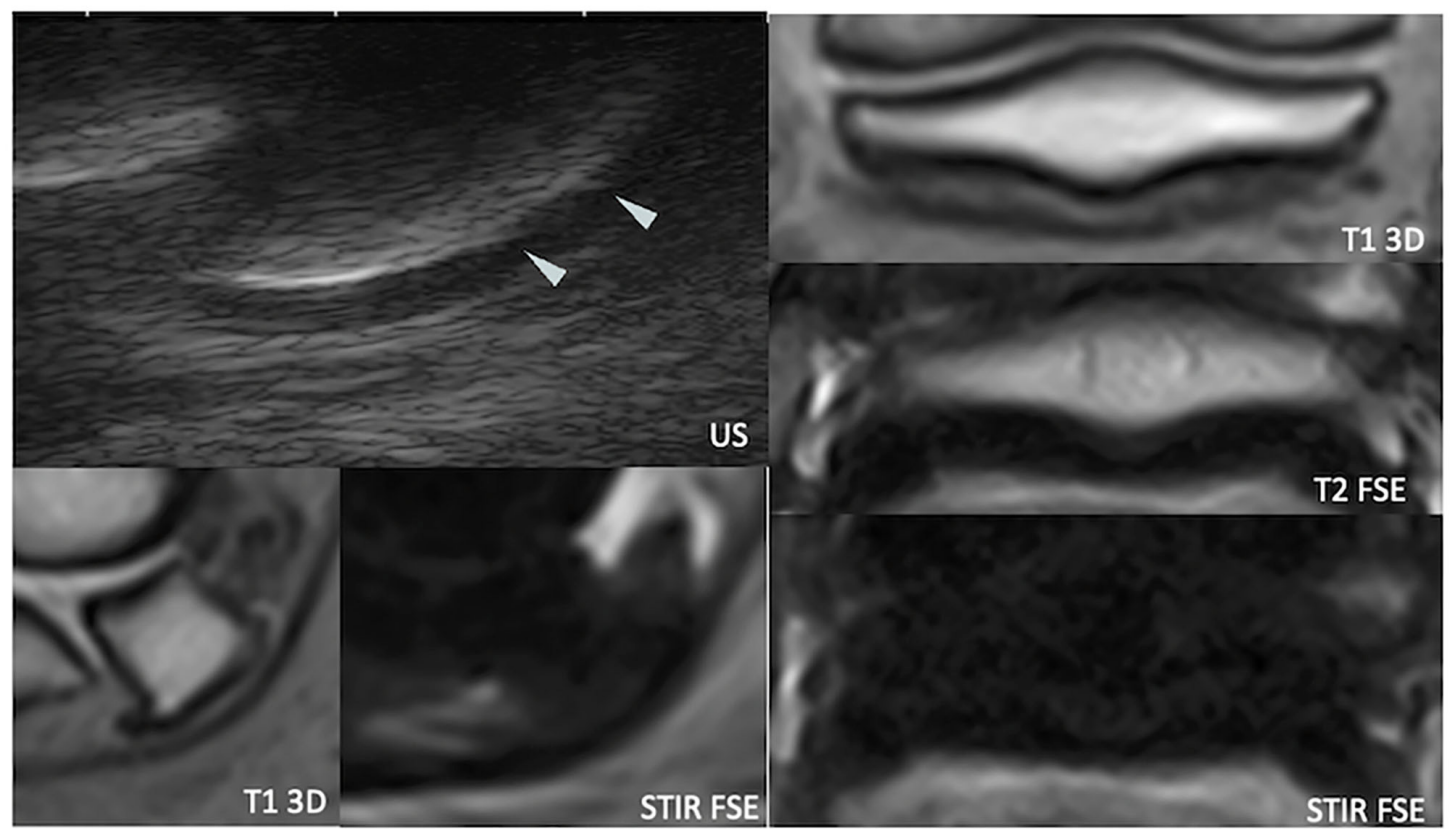
Longitudinal transcuneal ultrasonographic (top left), sagittal (bottom left), and transverse (right) magnetic resonance (MR) images of a right front foot, focused on the navicular bone. The ultrasonographic image has been flipped vertically to obtain the same orientation than the MR images. The mid-sagittal fossa of the palmar navicular compact bone shows sharp ultrasonographic margins (arrowheads). US, ultrasonography; T1 3D, tridimensional gradient echo T1 weighted sequence; T2 FSE, fast spin echo T2 weighted sequence; STIR FSE, fast spin echo short tau inversion recovery weighted sequence.

Abnormalities of the palmar aspect of the navicular bone were observed in 23 feet at ultrasonography and in 30 feet at sMRI (Figures 4–6). Standing magnetic resonance imaging allowed to identify abnormalities of the navicular spongiosa in 24 feet, which was obviously not evaluable with ultrasonography.

Ultrasonography identified diffuse irregularity of the flexor surface (12 feet) or focal marginal irregularity of the mid-sagittal fossa (8 feet). Deep penetration of the ultrasonographic beam through an irregularly marginated flexor surface characterized palmar compact erosions (three feet).

Standing magnetic resonance imaging identified palmar compact erosions (four feet) (with osseous cyst-like lesion formation in one case) and irregularity of the palmar compact without deep defect (six feet). The palmar fibrocartilage subjectively appeared heterogeneous and/or thin in all of these cases, as well as in 15 further feet.

The deep palmar compact erosions were detected with both modalities in three feet, whereas ultrasonography did not detect a distolateral defect visible at sMRI in the fourth foot. All but two feet with an irregular flexor surface or a sharp synovial fossa at ultrasonography had an irregular chondro-osseous margin or thin and/or heterogeneous fibrocartilage at sMRI.

Percentage of agreement for detection of palmar navicular changes (excluding the bone edema-like lesions of the palmar spongiosa) was 63.6%. Cohen's kappa indicated slight agreement (k = 0.14), but agreement was fair when adjusted for prevalence and bias agreement (PABAk = 0.27).

#### Navicular Bursa

**Figure 7:**
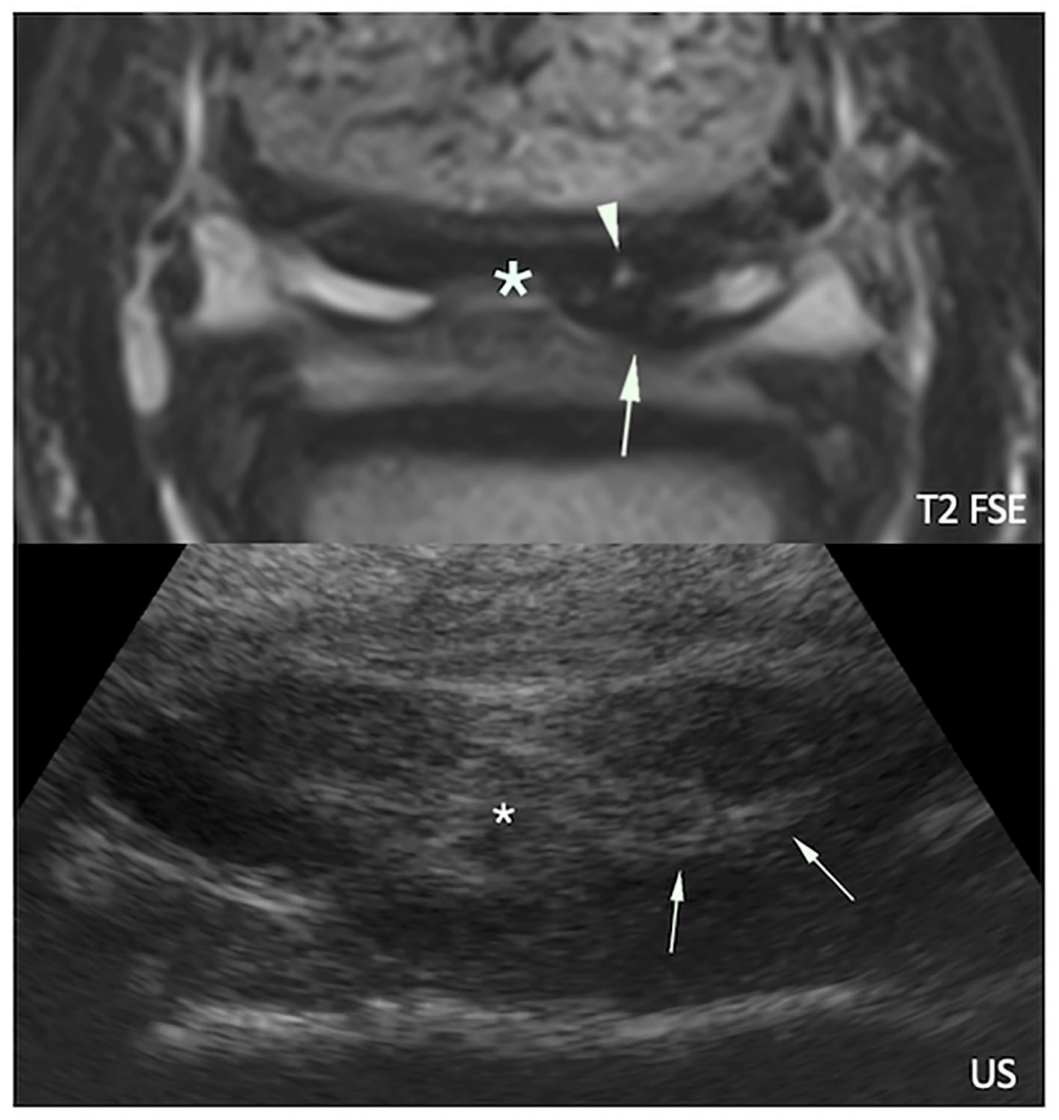
Transverse magnetic resonance (MR, top) and ultrasonographic (bottom) images of a right front foot focused on the deep digital flexor tendon (DDFT) and navicular bursa: lateral is to the right and dorsal to the bottom. The navicular bursa is distended by a moderate amount of fluid, and there is marked thickening of the mesotendon (asterisks). Proliferated soft tissue material (arrows) is visible adjacent to a MR hyperintense dorsal lesion of the DDFT lateral lobe (arrowhead).

Standing magnetic resonance imaging reported more abnormalities of the navicular bursa than ultrasonography, mainly seen as fluid effusion with or without soft tissue signal intensity material and/or mesotendon thickening (Figure 7). Both ultrasonography and sMRI detected bursa effusion in 20/34 feet, whereas 7/34 feet were considered not effused neither with ultrasonography nor with sMRI.

A discrepancy between sMRI and ultrasonographic detection of bursa effusion was seen in 7/34 feet (six feet detected with sMRI only, one foot with ultrasonography only). Occasionally soft tissue material was seen without associated fluid effusion (one foot at ultrasonography and four feet at sMRI). Adhesions were suspected on the attenuation of the synovial fluid signal by clustered or band shaped T2W hypointense material between the DDFT and CSL at sMRI (9 feet), and on the presence of echogenic soft tissue material replacing anechoic fluid at the same location at ultrasonography (five feet).

Percentage of agreement for detection of the navicular bursa effusion and/or proliferation was 70.6%. Cohen's kappa indicated fair agreement (k = 0.24), but agreement was moderate when adjusted for prevalence and bias (PABAk = 0.41).

## Discussion

This study compares the presence and appearance of lesions of the DDFT, navicular bone, and navicular bursa at ultrasonography and sMRI. In the present study, most abnormalities were identified by both modalities; however, agreement was relatively low. Ultrasonography suspected more DDFT abnormalities than sMRI. Defects of the palmar compact bone were identified with both modalities except a parasagittal defect, which was only seen at sMRI. Changes in echogenicity of the DDFT did not correspond to a particular pattern of sMRI signal change, and ultrasonography did not allow the assessment of morphology and extent, neither to discriminate the affected lobe of distal DDFT lesions in comparison to sMRI.

Earlier studies have reported MRI or ultrasonographic appearance of these structures; however, studies directly comparing sMRI and ultrasonographic findings are sparce (12, 37). The strength of this study is that it is prospective and that in each modality image acquisition and interpretation have been performed by the same operators, blindly and independently of the results obtained in the other modality. On the other hand, this study design has produced a relatively small number of horse's owned clients to be included in a busy clinical setting as both operators had to be available for the exam at the time of presentation. Population size is therefore relatively small, and this reduces the power of kappa statistics. Indeed, sample size requirement for a significant kappa of 0.4 and for the positive agreement found in the present study would be higher than 31 horses (36). Prevalence-adjusted and bias-adjusted kappa is proposed to consider influence of prevalence and bias, respectively, balancing k when chance agreement is high because of very high or very low prevalence of an abnormality and when unbalanced marginal totals produce higher values of k than more balanced totals but do not overcome sample size. Comparison of k to PABAk may give an indication of the likely effects of prevalence and bias in the study context for each specific abnormality. However, because of the reduced statistical power, the study is mainly useful to illustrate in which cases combination of imaging techniques may be indicated and how ultrasonography may be useful for staged imaging (although less skilled operators may do not obtain identical results because of the high technicality of foot ultrasonography).

Considering the total number of lesions detected in the present study, prevalence of DDFT abnormalities was higher than in previous reports (3, 18, 21). This may be partially explained by selection of horses responding to a palmar digital diagnostic analgesia (either sesamoid abaxial or distal digital) and the exclusion of horses with relevant radiographic abnormalities despite the clinical need of a sMRI examination to further complete assessment of the foot. This exclusion criterium was decided to avoid confirmation biases while acquiring and reading images in the other modalities as at least one of the operators would have seen the horse's radiographs prior to sMRI or ultrasonography. This selection criteria may also explain the overrepresentation of soft tissue lesions in comparison to navicular bone abnormalities. High prevalence of DDFT abnormalities may also be related to hospital population including mainly pleasure horses and low-level sport horses. Finally, because the study was not aimed to consider clinical significance of lesion but only image abnormalities, an overrepresentation of minor, non-clinically significant, DDFT lesions is possible in comparison to more clinically oriented studies.

The present study hypothesized that suprasesamoidean DDFT lesions observed at sMRI will be visible at ultrasonography. Considering the presence or absence of suprasesamoidean DDFT lesions, ultrasonography had a tendency to overestimate the presence of suprasesamoidean DDFT lesions in comparison to sMRI. In the absence of a gold standard (post-mortem or high field MRI), it is impossible to know if ultrasonographic or sMRI findings are, respectively, false positive or false negative results. Because the impression of a change in shape or size of a lobe can be induced by obliquity of the section at ultrasonography, and the good correlation reported for DDFT lesions between sMRI, high field MRI, and gross pathology (29), this result likely represents a real overestimation of positive cases by ultrasonography. On the other hand, sMRI may in some cases underestimate tendon involvement because of lack of delineation from the thickened adjacent soft tissue material in the bursa or by its limited spatial resolution (38).

Dorsal suprasesamoidean lesions were the most represented tendon abnormalities detected with both modalities. This is in accordance with previous results (17, 21, 33, 39, 40). However, ultrasonography did not allow to discriminate simple dorsal lesions from complex lesions with concomitant core lesions and/or with marked disruption of the dorsal tendinous border. Because it has been postulated that dorsal lesions carry a better prognosis than core lesions and splits (41, 42), and because treatment of dorsal lesions at bursoscopy is suggested in some hospitals (39), lesion characterization by MRI should be suggested for optimized patient management. This is particularly relevant for sport horses and/or if there is a lack of response to medical treatments.

Standing magnetic resonance imaging allowed detection of DDFT lesional fluid component, with the fluid sensitive spin echo sequences. These sequences and more particularly the STIR FSE sequence previously demonstrated a good ability to detect core necrosis associated with chronic degenerative tendon lesions (30). Other lesional tissue alteration with an increased fluid content (i.e., hemorrhage or oedema) may show similar signal hyperintensity on FSE sequences. Conversely, echogenicity at ultrasonography did not appear to be related to any particular MRI type or severity of lesion in the distal DDFT. In the distal digit, the oblique incidence of the ultrasonographic beam is responsible for overall hypoechogenicity of the tendon lobes in the suprasesamoidean area, which is related to their anisotropic properties (2, 4). Although markedly hypoechoic lesions may become more conspicuous and appear more hypoechoic than the remainder of the lobe when foot conformation allows a mild obliquity of the beam, ultrasonography may have some limitations in its ability to monitor the evolution of DDFT lesion over time. In contrary, the evolution of the MRI signal with time has demonstrated its value as prognostic factor and helping with long-term patient management (41).

Ultrasonography allowed identification of suprasesamoidean lesion vascularization in four cases, where a strong Doppler signal was observed associated with the lesion. The use of intra-venous and intra-arterial administration of contrast media in the equine foot during MRI examination under general anesthesia has recently been validated, allowing to highlight vascularization of DDFT lesions, adhesions, navicular spongiosa, and peritendinous tissues (43, 44). However, intra-venous or intra-arterial contrast is not yet routinely used during MRI examinations, and for practical reasons was not applied to our patients. In light of the fact that Doppler signal has been suggested to be correlated to pain, severity, and age of the lesions (45, 46), power Doppler may be a useful adjunctive diagnostic test when suprasesamoidean lesions are detected ultrasonographically.

Ultrasonography also allowed prompt and easy detection of lesions in the proximal pastern. This region was not included in the primary field of view of the foot examination in sMRI as it would require the acquisition of additional sequences with the coil located more proximal and this is not the routine practice in our clinic. Therefore, this study does not include direct comparison of the sMRI and ultrasonographic appearance of proximal pastern DDFT lesions. However, given the relative high number of proximally extending lesions, ultrasonography represents a useful adjunct to sMRI and should be used to rapidly and easily extend the area of assessment of DDFT damage and detect proximal lesions, important for future management.

Mineralization was suspected at ultrasonography in three feet. Secondary to their very short T2 relaxation times, both the tendon and the calcified material have very low signal intensity on conventional MRI sequences (47, 48), and therefore, intratendinous mineralization may remain undetected at MRI, as it was the case in the present study. However, in 2/3 feet with lesion mineralization observed at ultrasonography, severe sMRI lesions were identified despite mineralization was not seen.

The present study hypothesized that sesamoidean DDFT lesions observed at sMRI would not be identified with ultrasonography, while infrasesamoidean DDFT lesions would be seen at transcuneal ultrasonography only in some cases if the lesion is severe enough to produce a change of the palmar profile of the tendon. In the sesamoidean region, the decreased tendon thickness may be responsible for a lower ability of MRI to detect lesions due to their small size in relation with the spatial resolution of the technique, more particularly for the standing low-field system. Therefore, the sesamoidean portion of the DDFT remains a challenging area to evaluate, regardless of chosen technique. Transcuneal ultrasonography is limited by the frog conformation and the hydration status of the hoof tissue, which may be breed dependent (5). It is also limited by the impossibility to adequately image the abaxial portions of the tendon because of the sagittal acoustic window allowed by the frog (5). Furthermore, the assessment of the most axial parts of the DDFT lobes at ultrasonography is limited to the use of a slightly oblique probe angle (5, 10). Because of this, ultrasonography was unable to lateralize the affected lobe in most cases where the lesion was detected. In three cases with larger lesions, it was, however, possible to characterize the palmar tendon deformation or change in echogenicity as being more visible laterally, which corresponded with the sMRI location.

The most represented sesamoidean tendon sMRI abnormalities were dorsal border irregularities, which is in accordance with previous studies (17, 39). These dorsal border irregularities were undetected with ultrasonography, due to imperceptible delineation between the tendon dorsal border and the palmar navicular bone fibrocartilage (5). Conversely, ultrasonography was able to detect tendon thickening in distal sesamoidean and infrasesamoidean areas. However, ultrasonography was not able to discriminate core from splits or dorsal lesions as sMRI did. Deformity of the palmar profile of the tendon has already been reported as the only significant change visible at ultrasonography (7) and explains the limitation of the technique in this particular area.

The hypothesis that abnormalities of the flexor surface of the navicular bone will be visible both at sMRI and at transcuneal ultrasonography was confirmed. However, sMRI detected more fibrocartilage changes in comparison to ultrasonography. Ultrasonography and sMRI have a good sensitivity to detect abnormalities of the bone surface (49, 50). Low-field sMRI has limited spatial resolution (38) but presents the advantage of providing a more thorough assessment of the palmar fibrocartilage, palmar compact, and spongiosa, including changes such as bone edema-like lesions. Thinning and heterogeneous signal intensity of the palmar fibrocartilage have been suggested as signs of low-grade abnormality of the flexor surface of the navicular bone on high-field MRI (25). However, non-contrast MRI has demonstrated a poor accuracy in detecting fibrocartilage damage (39, 51), and a sensitivity and a specificity of 100 and 6%, respectively, of high-field MRI compared with histology (51). The limited spatial resolution of low-field sMRI further limits the accuracy of our results in comparison to high-field MRI studies, and at the same time, a certain “pressure to succeed” may have influenced the false positive rates even in standing low-field sMRI as considered the technique of choice to assess the equine foot in clinical conditions.

As expected, the hypothesis that the lesions of the spongiosa of the navicular bone identified with sMRI as bone edema-like lesions would not be visible at ultrasonography was confirmed.

Navicular bursa effusion and soft tissue material were diagnosed more frequently with sMRI than with ultrasonography. Chronic bursitis with increased soft tissue content might be considered in cases with advanced dorsal tendon pathology without visible effusion of the navicular bursa with ultrasonography. Regarding fluid effusion, pressure on the probe in some cases can lead to an iatrogenic decrease in size of the proximal palmar recesses, especially if the examination is performed on a non-weight-bearing limb. This may reduce the perception of subjective overdistension of the proximal recesses of the bursa in comparison to sMRI.

In the present study, sMRI was performed prior to ultrasonography, whereas in clinical conditions, it would be done after to follow a rational staged-imaging process. This particular order was chosen to optimize the amount of sedation administered to the horse and, although non-representative of clinical conditions, was not considered to influence the results of the study since ultrasonographic examiner was blinded to the sMRI results. The absence of a control group could have influenced the results as both the ultrasonographer and sMRI operator were aware of the clinical status of the cases. Performing blinded ultrasonographic and sMRI examinations on control horses was, however, not considered feasible as the operators were involved in the patient selection process.

Finally, it has to be recognized that in the absence of a gold standard as histopathology, no accuracy, specificity, sensitivity, positive predictive value, and negative predictive value have been calculated in the present study. This was considered to be beyond the objective of this study, which aimed to compare the relative appearance of the lesions detected by both modalities and the agreement between the two modalities in real clinical conditions. Further comparative studies with post-mortem gold standards, which are for obvious reasons not feasible in clinical conditions, are needed to assess real false positive and false negative results in each modality.

In conclusion this study describes the appearance of lesions detected by ultrasonography and sMRI in a series of clinical cases. The findings indicate the strengths and limitations of each technique, further depicting how they can complement each other, and, given the better agreement between sMRI and ultrasonography for the suprasesamoidean DDFT lesions, suggest that ultrasound may be used as a screening for these lesions, when MRI is not feasible for geographical or financial reasons or before a complete MRI assessment. The presence of ultrasonographic abnormalities will not preclude the use of sMRI because of the better characterization of morphology, extent and fluid content of DDFT lesions, and the ability of sMRI to better assess distal DDFT and detect concurrent changes, in particular in the navicular spongiosa.

## Data Availability Statement

The raw data supporting the conclusions of this article will be made available by the authors, without undue reservation.

## Author Contributions

LE contributed to conception, design and drafting of the article, acquisition, analysis, and interpretation of data. ZJ and MV contributed to data acquisition and interpretation, as well as revising the article for intellectual content. FA contributed to conception and design of the article, data analysis and interpretation, and revising the article for intellectual content. VB contributed to conception and design of the article, acquisition, analysis, and interpretation of data, and revising the article for intellectual content. All authors contributed to the article and approved the submitted version.

## Conflict of Interest

The authors declare that the research was conducted in the absence of any commercial or financial relationships that could be construed as a potential conflict of interest.
