# Supplementary material for: Comparison Between Ultrasonographic and Standing Magnetic Resonance Imaging Findings in the Podotrochlear Apparatus of Horses With Foot Pain
Source: Front Vet Sci. 2021 Jul 5;8:675180. doi: 10.3389/fvets.2021.675180 (PMC8287171; doi:10.3389/fvets.2021.675180)
Supplement: Supplementary file 1 [file Table_1.DOCX]

**Supplementary Table 1.** Summary and distribution of deep digital flexor tendon lesions observed at ultrasonography and standing magnetic resonance imaging.

| **Area** | **US** | | | | | **sMRI** | | | | | **Lesions detected** **US & sMRI**  (n = feet) |
| --- | --- | --- | --- | --- | --- | --- | --- | --- | --- | --- | --- |
|  | Tot. feet | Tot. lobes | | Lesion morphology  (n = feet) | | Tot. feet | Tot. lobes | | Lesion morphology  (n = feet) | |  |
| **Pastern** | 12 | Medial  Lateral  Axial | 6  7  1 | Dorsal  Core  Palmar  Axial thickening | 6  3  2  1 | NA | NA | | NA | | NA |
| **Suprases** | 30 | Medial  Lateral | 18  25 | Dorsal  Thickening  Full-thickness hyperechoic line | 27  2  1 | 24 | Medial  Lateral | 16  21 | Dorsal border lesion  *Including complex lesions*  Dorsal irregularity/deformity without signal change | 19  6  5 | 22 |
| **Ses** | 20 | Lateral  NL | 3  17 | Tendon thickening /palmar convexity | 17 | 17 | Medial  Lateral | 9  14 | Dorsal irregularity + core  Dorsal irregularity  Split  Split + core  Core | 7  4  4  1  1 | 15 |
| **Infrases** |  |  |  | Lateral palmar convexity  Lateral hypoechogenicity | 2  1 | 12 | Medial  Lateral | 7  5 | Split  Core  Dorsal | 5  5  2 |  |

US: ultrasonography ; sMRI: standing magnetic resonance imaging; Suprases : suprasesamoidean level ; Ses : sesamoidean level ; Infrases : infrasesamoidean level ; tot. feet : total number of affected feet ; tot. lobes: total number of affected lobes ; NL: non-lateralized; NA: non applicable ;

* complex lesions: extensive lesions combining dorsal lesion and core or split
